# Acute and Subacute Toxicity Assessment of Oxyclozanide in Wistar Rats

**DOI:** 10.3389/fvets.2019.00294

**Published:** 2019-09-06

**Authors:** Weiwei Wang, Zhen Dong, Jili Zhang, Xuzheng Zhou, Xiaojuan Wei, Fusheng Cheng, Bing Li, Jiyu Zhang

**Affiliations:** ^1^Key Laboratory of New Animal Drug Project of Gansu Province, Lanzhou, China; ^2^Key Laboratory of Veterinary Pharmaceutical Development, Ministry of Agriculture, Lanzhou, China; ^3^Lanzhou Institute of Husbandry and Pharmaceutical Sciences, Chinese Academy of Agricultural Sciences, Lanzhou, China

**Keywords:** oxyclozanide, acute, subacute, toxicity, rats

## Abstract

Oxyclozanide is an effective anthelmintic and has shown good properties in other ways including anti-adenovirus, anti-biofilm, antifungal, and antibacterial activity. This study aimed to investigate the acute and subacute 28-days repeated dose oral toxicity of an oxyclozanide suspension in Wistar rats. A high oral lethal dose (LD_50_) of 3,707 mg/kg was observed in the acute toxicity test. During the 28-days time period, no obvious adverse effects or death were detected. Histopathological changes were observed in the heart, liver, and kidney of animals treated with high dose of oxyclozanide. Based on the hematological parameters, there were no statistical differences between the oxyclozanide-treated group and the control group. For biochemistry assays, ALP, AST, GLU, TBIL, GLO, TG, BUN, UA, LDH, and CK were statistically changed in the treatment groups. These data suggested that the LD_50_ of oxyclozanide was ~3,707 mg/kg body weight (BW), and the lowest observed adverse effect level (LOAEL) of oxyclozanide was at a dose of 74 mg/kg in rats.

## Introduction

Infectious parasitic diseases have become a serious threat to animal health and productivity in developing countries (1–3). Among them, fascioliasis, as a zoonotic disease, is a food-borne parasitic disease, which is transmitted to certain hosts, humans, and other herbivorous mammals through contaminated water or green vegetables (4). It is also considered to be one of the most serious neglected tropical diseases. The pathogens of fascioliasis are *Fasciola hepatica* as well as *Fasciola gigantica*. *F. hepatica* is an important tissue parasite, which can cause serious pathological damage to the host and have a great impact on animal husbandry. It is estimated that the annual loss of global animal production caused by *F. hepatica* infection is more than $3.2 billion. In addition, *F. hepatica* is considered to be a new infectious disease in human beings. The World Health Organization estimates that 180 million people are at risk of infection and 2.4 million are infected with fascioliasis (5).

Since no vaccine has been successfully developed, the effective strategy for controlling fascioliasis depends mainly on the use of pharmaceuticals (fasciolicide). Oxyclozanide was developed in 1966 (6) and has achieved an effective effect in subsequent clinical applications (7, 8). In clinical trials, oxyclozanide has been found to be effective not only for the treatment of *F. hepatica* infection but also for other tissue parasites, such as *Hymenolepis* sp. (9) and *Paramphistomum* sp. (10).

Compared with other fasciolicides, such as closantel, albendazole, and clorsulon, oxyclozanide is the most effective drug in ruminants naturally infected with *F. hepatica* (11–13). According to Rana's recent review, oxyclozanide was effective on mature fluke, especially for adults over 14 weeks old (14). Additionally, a resistance of triclabendazole and albendazole in sheep, cattle, and water buffalos was reported widely (15–19). Fortunately, oxyclozanide resistance has still not been reported in animals and has a very good killing effect on *F. hepatica* resistant to triclabendazole (20). Oxyclozanide has also good pharmacokinetic characteristics, including rapid absorption and long elimination of half-life (10, 21, 22).

In recent years, oxyclozanide has still been explored and researched. The researchers found that oxyclozanide showed anti-adenovirus activity and almost no cytotoxicity at low concentration and proved its mechanism as a specific target to inhibit the transcription of early adenovirus gene E1A (23). Maiden and co-workers defined oxyclozanide as an anti-biofilm agent that enhances the activity of aminoglycosides and tetracycline against *Pseudomonas aeruginosa* biofilm by reducing membrane potential, penetrating cells, and enhancing accumulation of tobramycin in biofilm (24). Oxyclozanide also showed excellent antifungal activity and antibacterial activity at low concentrations (25, 26). It reported that the uncoupling of mitochondria could reduce the formation of intestinal polyps and the metastasis of colon cancer cells (27).

Those studies have attracted much more attention for the application of oxyclozanide. Thus, the toxic evaluation of oxyclozanide for clinic use seems to be important and need to be further studied. Although the study of oxyclozanide has already begun broadly in the 1960s, the toxicity assessment is still less known. The acute and 28-day repeated oral toxicity of oxyclozanide were carried out to supply the toxicity of oxyclozanide and to provide guidance for phase II clinical trials. In this study, we evaluated the acute and subacute toxicity of oxyclozanide in rats for oral administration.

## Materials and Methods

### Ethics Statement

All animal studies were performed according to the US National Institutes of Health Guidelines for the Care and Use of Laboratory Animals and approved by the Institutional Animal Care and Use Committee of Lanzhou Institute of Husbandry and Pharmaceutical Science of Chinese Academy of Agricultural Sciences. The animals were examined and adapted to the new environmental conditions for a week before the formal experiment.

### Oxyclozanide Suspension

3,3′,5,5′,6-Pentachloro-2,2′-dihydroxybenzanilide (oxyclozanide) is a white crystalline solid, non-hygroscopic, and virtually insoluble in water (7). Oxyclozanide was purchased from Jiangsu Quality Horizons Pharmtech Incorporation. The purity of the drug was 98.76%, which was assessed by high-performance liquid chromatography (HPLC, Agilent, USA) in our laboratory. For the physical properties of the drug, it was mixed with 0.2% sodium carboxymethyl cellulose solution. The concentrations of oxyclozanide suspension were 250.00, 184.50, 136.50, 101.25, and 75.00 mg/mL. For the acute study, 20 rats were divided into five groups (two males, two females) in pre-experiments. The high-dose group was 5,000 mg/kg and the low-dose group was 1,000 mg/kg. After dosing, according to Veterinary Drug Research (28), rats were observed and recorded for 7 days. Animal death was recorded. In the formula, “*a*” referred to LD_0_, “*b*” referred to LD_100_. *N* (group number), and *r* (ratio of dosage from each group) were calculated according to Koch's Method (*N* = *b*/*a*; *r* = lg – 1[(lg*b* – lg*a*)/(*N* – 1)]). Based on the results of pre-experiments, 60 rats were divided into six groups. The treated group received oxyclozanide at the dosage of 5,000, 3,690, 2,730, 2,025, and 1,500 mg/kg BW/day. For the subacute study, according to Veterinary Drug Research (28), one-tenth to one-fourth of LD_50_ is the high dose daily administration for 28 days. In the 28-day test, one-tenth is chosen. In the medium-dose group, one-half of the high dose is medium dose. In the low-dose group, one half of medium dose is low dose. The concentrations of oxyclozanide suspension were 55.0, 27.8, and 11.1 mg/mL.

### Animals

Healthy male and female Wistar rats (100–150 g) were purchased from the Laboratory Animal Center of Lanzhou Veterinary Research Institute [Certification No. SCXK (G) 2015-001]. The toxicity test was performed strictly with the method of Compilation of Technical guidelines for Veterinary Drug Research (28) and was also referred to Organization for Economic Cooperation and Development (OECD) Guideline No. 407 (29). All animals were housed in plastic cages and kept separately according to sex (temperature 22 ± 2°C, relative humidity 55 ± 10%, and 12-h light/dark cycle). All rats were allowed to access water and food freely.

### Acute Toxicity Experiment

Sixty rats (8 weeks old) were used in this experiment and were randomly assigned into six groups (five males and five females). The control group received 0.2% carboxymethyl cellulose sodium solution in the same volume, while the treated group received oxyclozanide at the dosage of 5,000, 3,690, 2,730, 2,025, and 1,500 mg/kg BW/day. After 7-day acclimatization, rats were treated once. The general clinic observations will start after rats were given drugs 4 h later and then this record will last for at least 7 days every 8 h.

### Subacute Toxicity Experiment

Ninety-six rats (6 weeks old) were randomly assigned into four groups (12 male and 12 female rats in each group). The obtained median lethal dose (LD_50_) of oxyclozanide was 3,707 mg/kg BW/day in rats. The four groups were divided into: high dose (370 mg/kg BW/day), medium dose (185 mg/kg BW/day), low dose (74 mg/kg BW/day), and control (0.2% carboxymethyl cellulose sodium solution in same volume). Rats were administered once daily by gavage with oxyclozanide suspension at three doses as above (370, 185, and 74 mg/kg BW/day) at 2:00–3:00 p.m. throughout the experiment for 28 continuous days. Hold the animal with the left hand and the syringe with the right hand. Keep the head and neck of the rat in a straight line, so that the needle can enter the mouth easily. In Baoding rats, the head must be fixed well to avoid the head twisting at will and affect the operation of gavage. The intragastric needle enters from the angulus oris of the animal to make the mouth and esophagus align. When the intragastric needle enters about 5 cm deep, gently push the syringe to make the drug enter the rat body slowly. If there is no excessive struggle, we can try to inject drugs slowly. If the resistance is small, we can inject all drugs. In the case of rat struggle, the intragastric needle should be pulled out, reserved and operated again. The animals' general clinic observations, body weight, morbidity, and mortality were recorded daily during administration. Additionally, measurements of food consumption were recorded weekly.

#### Hematological Index

Blood samples were collected and placed into tubes containing EDTA-K_2_ for the hematological analyses. At the end of the drug administration period, hematological index of rats was analyzed. Standard hematological and biochemistry tests were used to determine the hematological parameters, enzymes, substrates, and products of metabolism. The following indicators were red blood cell count (RBC), white blood cell count (WBC), lymphocyte count (LYM), monocyte count (MON), granulocyte count (GRAN), hemoglobin (HGB), hematocrit (HCT), mean corpuscular volume (MCV), mean corpuscular hemoglobin (MCH), mean corpuscular hemoglobin concentration (MCHC), red blood cell distribution width (RDW), platelet count (PLT), platelet distribution width (PDW), mean platelet volume (MPV), and thrombocytocrit (PCT).

#### Blood Biochemical Index

Blood samples were collected, centrifuged at 4,000 rpm for 10 min at 4°C and stored at −20°C. At the end of the drug administration period, blood biochemical index of rats in each group was analyzed. The main biochemical indicators were alanine aminotransferase (ALT), aspartate aminotransferase (AST), alkaline phosphatase (ALP), urea nitrogen (BUN), total bilirubin (TBIL), direct bilirubin (DBIL), creatinine (Cr), total protein (TP), blood sugar (Glu), serum albumin (Alb), total protein (TP), total cholesterol (TCH) and triglyceride (TG), globulin (GLO), total bile acid (TBA), glucose (GLU), TG, total cholesterol (TC), creatine kinase (CK), lactate dehydrogenase (LDH), and uric acid (UA).

#### Necropsy

After 28-day drug administration, treated and untreated rats were euthanized and were then necropsied. Necropsies were conducted carefully on all the rats, which either died or survived during experiments. Tissues like heart, liver, spleen, lungs, kidney, brain, reproductive organs (tests, ovary and uterus), prostate, gland, adrenal gland, white adipose tissue, thyroid, thymus, stomach, urinary bladder, intestine, pancreas, bones, and peripheral nerves were examined for the macroscopic morphology and then removed quickly, washed twice with ice-cold saline, weighed, and kept in AAF fixation solution (Solarbio, Beijing, China) for at least 48 h. In addition, the relative weight of each organ (viscera/body ratio) was calculated based on the animal's body weights according to the following formula: organ weight/body weight on sacrifice day × 100, as described previously (30).

#### Histopathological Examination

Tissues were dehydrated in ethyl alcohol and treated with ethyl salicylate. After the tissue was embedded in paraffin and sectioned at the 5-μm thickness, sections were dyed with hematoxylin and eosin (H&E) according to the universally accepted procedure for light microscopy.

### Statistical Analysis

In the toxicity study, data were expressed as mean value and standard deviations (mean ± SD). Statistical analyses containing body weight, organ weight, blood biochemical, and biochemistry data were performed by one-way analysis of variance (ANOVA) and Levene's test for homogeneity of variance with SPSS Statistics 19.0 (IBM, Chicago, USA), and the graph was drawn by using GraphPad Prism 5.0 (GraphPad Prism, San Diego, USA). A *p* < 0.05 was considered statistically significant.

## Results

### Acute Toxicity

In the acute test, the toxic symptoms that were observed in rats included depression, rough hair, and inappetence in Groups 1, 2, 3, and 4. The lips, limbs, and tails of the dead rats were cyanotic. In addition, individual rats have swelling spleen, bleeding spots in the liver, and swelling stomachs in the high-dose group. Most of the animals died after administration during the first 12 h. The number of deaths during acute toxicity is shown in Table 1. Particularly, the death was mainly half male and half female, which illustrated that death has no relation to sex. Therefore, LD_50_ of oxyclozanide was 3,707 mg/kg BW/day for experimental rats in both sexes (95 confidence interval, 3,148–4,365 mg/kg BW/day), which was calculated by the Koch's method (31) (Table 1).

**Table 1:** The death of rats during the acute toxicity study.

**Group**	**Number dosed**	**Dosage (mg/kg)**	**Mortality rates (%)**	**Deaths (*n*)**	**Survivals (*n*)**
1	10	5,000	100%	10	0
2	10	3,690	40%	4	6
3	10	2,730	20%	2	8
4	10	2,025	10%	1	9
5	10	1,500	0%	0	10

### Subacute Toxicity

#### Morphological Alternation

No treatment-related deaths occurred during the 28-day test. Clinic general observations showed that there was no apparent alteration in general signs like posture, gait, and response to handling, but slight depression and diarrhea were detected in the high-dose group during administration. Furthermore, oxyclozanide hasn't changed the skin, fur colors, eyes, mucous membrane, the occurrence of secretions and excretions, motor activities, and autonomic activity.

#### Assessment of Food Consumption and Body Weight

Food consumption was recorded but no statistical alternation in food consumption (data not shown), illustrating that the oxyclozanide has no obvious effect on food consumption.

**Figure 1:**
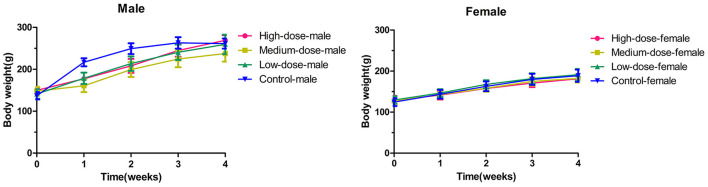
The changes of body weight in rats during 28 days (4 weeks) with oxyclozanide treatment at 0 (control), 74 mg/kg BW/day (low dose), 185 mg/kg BW/day (medium dose), or 370 mg/kg BW/day (high dose) group.

The mean values of the body weight gain in the high-dose group and the medium-dose group were lower than those in the control group (*p* < 0.05 for male rats in the medium-dose group and female rats in the high-dose group vs. their representative controls). However, no statistical changes (*p* > 0.05) were observed between the low-dose group and the control group (Figure 1).

#### Hematology Index

All the relative blood parameters, including Lym, Mon, WBC, HBC, and PCT, were not statistically changed between the oxyclozanide-treated group and the control group, except MCH levels in both male and female rats, and MCHC, RDW, and PCT levels in female rats vs. their respective controls (Table 2). In particular, the statistical findings occurred in the low-dose group. These statistical changes were still in the range of historical controls. Deep reasons of differences need to be further studied.

**Table 2:** Haematological values of rats in oxyclozanide-treated group and control group.

	**Historical controls range (45)**		**Oxyclozanide doses (mg/kg)**		
		**0 mg/kg (control)**	**74 mg/kg (low-dose)**	**185 mg/kg (medium-dose)**	**370 mg/kg (high-dose)**
**Male**					
WBC (× 10^9^·L^−1^)	4–6	5.28 ± 2.12	4.00 ± 1.73	5.47 ± 2.44	5.13 ± 1.22
LYM (× 10^9^·L^−1^)	2–5	2.87 ± 1.70	3.50 ± 1.22	4.10 ± 2.01	3.88 ± 0.96
Mon (× 10^9^·L^−1^)	0.05–0.10	0.05 ± 0.071	0.06 ± 0.074	0.08 ± 0.071	0.08 ± 0.044
Gran (× 10^9^·L^−1^)	0.5–1.0	0.54 ± 0.46	0.63 ± 0.43	0.80 ± 0.48	0.98 ± 0.48
RBC (× 10^12^·L^−1^)	6–8	7.04 ± 0.38	7.29 ± 0.51	7.03 ± 0.48	6.93 ± 0.45
HGB (g·L −1)	120–140	137.55 ± 5.47	128.60 ± 22.83	135.38 ± 10.21	136.17 ± 10.53
HCT	35–45	41.57 ± 1.50	43.14 ± 3.10	41.26 ± 3.02	41.18 ± 3.11
MCV (fL)	55–65	59.16 ± 1.91	59.24 ± 0.97	58.75 ± 1.88	59.47 ± 2.32
MCH (pg)	17–21	19.51 ± 0.48	17.37 ± 3.01^**^	19.18 ± 0.46	19.58 ± 0.67
MCHC (g·L^−1^)	270–340	330.36 ± 4.39	293.67 ± 50.54	327.50 ± 7.44	330.17 ± 7.98
RDW	10–15	11.60 ± 2.21	10.84 ± 1.69	12.75 ± 1.84	12.60 ± 1.81
PLT (× 10^9^·L^−1^)	800–1,000	860.18 ± 153.11	939.44 ± 154.04	956.38 ± 68.66	759.40 ± 144.87
MPV (fL)	5–7	6.38 ± 0.25	6.28 ± 0.31	6.09 ± 0.50	5.95 ± 0.66
PDW	15–17	16.19 ± 0.18	16.29 ± 0.32	16.13 ± 0.25	16.22 ± 0.43
PCT (ng·L^−1^)	0.4–0.8	0.53 ± 0.079	0.56 ± 0.073	0.58 ± 0.052	0.58 ± 0.090
**Female**					
WBC (× 10^9^·L^−1^)	4–6	4.14 ± 2.16	5.76 ± 1.63	4.66 ± 1.98	5.01 ± 1.36
LYM (× 10^9^·L^−1^)	3–5	3.46 ± 1.55	4.64 ± 0.612	4.25 ± 1.09	4.11 ± 1.14
Mon (× 10^9^·L^−1^)	0.05–0.10	0.09 ± 0.074	0.10 ± 0.076	0.08 ± 0.067	0.06 ± 0.052
Gran (× 10^9^·L^−1^)	0.6–1.1	0.86 ± 0.45	0.90 ± 0.41	1.00 ± 0.42	0.92 ± 0.20
RBC (× 10^12^·L^−1^)	6–8	7.45 ± 0.21	7.21 ± 1.31	7.26 ± 1.47	7.23 ± 0.35
HGB (g·L^−1^)	120–140	148.45 ± 7.85	126.38 ± 28.23	136.80 ± 38.84	143.88 ± 6.94
HCT	35–45	44.50 ± 1.22	42.80 ± 7.84	42.99 ± 8.63	43.70 ± 2.07
MCV (fL)	60 ± 6	59.86 ± 1.73	53.05 ± 19.12	59.30 ± 1.00	60.55 ± 2.37
MCH (pg)	17–21	19.89 ± 0.81	17.53 ± 2.35^*^	18.44 ± 3.49	19.88 ± 0.69
MCHC (g·L^−1^)	270–340	333.00 ± 15.50	296.13 ± 41.19^*^	311.78 ± 58.95	328.88 ± 4.12
RDW	10–15	14.92 ± 0.45	13.80 ± 1.16^**^	14.78 ± 0.55	14.41 ± 0.77
PLT (× 10^9^·L^−1^)	900–1,100	1,015.82 ± 91.63	970.38 ± 215.43	921.38 ± 192.32	953.33 ± 155.58
MPV (fL)	5–7	6.25 ± 0.22	6.33 ± 0.26	6.46 ± 0.69	6.18 ± 0.37
PDW	15–17	16.07 ± 0.13	16.13 ± 0.18	16.50 ± 1.15	16.10 ± 0.41
PCT (ng·L^−1^)	0.4–0.8	0.63 ± 0.056	0.55 ± 0.132^*^	0.49 ± 0.174	0.50 ± 0.197

* *p < 0.05 vs. control group*.

** *p < 0.01 vs. control group*.

#### Biochemical Analyses

Compared with the control group, biochemical analyses showed some statistical differences (*p* < 0.05) between oxyclozanide treatment and control group (Table 3). For instance, the levels of ALB, UA, LDH, and CK were statistically increased and GLU were decreased (*p* < 0.01 and *p* < 0.05, respectively) in the medium- and/or high-dose groups as compared with their respective controls only in male rats. In contrast, TBIL, GLO, and TG levels were statistically decreased (*p* < 0.01) in the medium- and/or high-dose groups as compared with their respective controls only in female rats. The levels of AST, ALP, and BUN were statistically elevated in the medium- and/or high-dose group vs. their respective controls in both male and female rats (*p* < 0.01 and *p* < 0.05, respectively).

**Table 3:** Liver function related to biochemical profiles.

	**Historical controls range (45)**		**Oxyclozanide doses**		
		**0 mg/kg (Control)**	**74 mg/kg (Low-dose)**	**185 mg/kg (Medium-dose)**	**370 mg/kg (High-dose)**
**Male**					
TBIL (μmol·L^−1^)	0.3–0.7	0.52 ± 0.23	0.54 ± 0.29	0.39 ± 0.19	0.35 ± 0.17
DBIL (μmol·L^−1^)	0.8–1.4	1.20 ± 0.55	0.97 ± 0.32	1.01 ± 0.22	1.32 ± 0.51
TP (g·L^−1^)	30–50	41.94 ± 15.15	36.17 ± 11.22	41.83 ± 9.38	49.97 ± 8.13
ALB (g·L^−1^)	18–26	19.45 ± 6.61	18.30 ± 5.53	19.63 ± 4.04	24.36 ± 3.48^*^
GLO (g·L^−1^)	16–26	22.45 ± 8.63	17.87 ± 5.73	22.20 ± 5.57	25.62 ± 4.90
ALT (U·L^−1^)	25–35	34.88 ± 12.47	27.35 ± 9.97	34.62 ± 9.68	32.45 ± 5.23
AST (U·L^−1^)	95–150	98.43 ± 33.86	132.14 ± 35.72	158.11 ± 48.63^**^	199.45 ± 32.55^**^
ALP (U·L^−1^)	120–200	131.71 ± 25.22	135.00 ± 26.93	197.00 ± 46.29^*^	224.00 ± 81.28^**^
TG (mmol·L^−1^	0.5–.07	0.62 ± 0.18	0.63 ± 0.17	0.68 ± 0.33	0.56 ± 0.16
TC (mmol·L^−1^	1.0–2.0	1.52 ± 0.47	1.34 ± 0.45	1.70 ± 0.36	1.70 ± 0.36
GLU (mmol·L^−1^)	4–8	6.64 ± 1.50	6.00 ± 1.15	5.49 ± 1.45	4.95 ± 1.09^**^
BUN (mmol·L^−1^)	5–9	5.49 ± 1.34	7.01 ± 1.98	7.17 ± 1.33	8.76 ± 2.99^**^
CR (μmol·L^−1^)	20–30	24.89 ± 17.67	21.05 ± 7.06	25.22 ± 5.19	29.46 ± 8.73
UA (μmol·L^−1^)	0.04–0.06	0.046 ± 0.012	0.045 ± 0.017	0.065 ± 0.021	0.080 ± 0.048^*^
LDH (U·L^−1^)	1,200–2,000	1,320.30 ± 469.50	1,582.11 ± 303.31	1,864.00 ± 433.37^**^	1,954.77 ± 236.24^**^
CK (U·L^−1^)	1,100–1,700	1,188.72 ± 488.39	1,210.48 ± 279.84	1,499.38 ± 405.02	1,628.94 ± 312.20^**^
**Female**					
TBIL (μmol·L^−1^)	0.4–0.8	0.69 ± 0.27	0.45 ± 0.26^*^	0.49 ± 0.14	0.27 ± 0.12^**^
DBIL (μmol·L^−1^)	1–2	1.32 ± 0.42	1.27 ± 0.47	1.46 ± 0.49	1.48 ± 0.43
TP (g·L^−1^)	40–48	45.85 ± 9.98	42.24 ± 10.40	45.40 ± 13.24	40.32 ± 4.21
ALB (g·L^−1^)	20–26	23.33 ± 4.65	21.60 ± 5.10	22.49 ± 6.09	25.87 ± 3.98
GLO (g·L^−1^)	20–30	22.52 ± 5.34	20.65 ± 5.36	22.91 ± 7.30	28.97 ± 6.49^**^
ALT (U·L^−1^)	25–35	26.11 ± 7.17	25.64 ± 10.56	30.68 ± 11.99	33.92 ± 6.11
AST (U·L^−1^)	130–180	137.10 ± 31.18	155.70 ± 39.79	177.71 ± 49.51^*^	206.60 ± 25.16^**^
ALP (U·L^−1^)	90–140	97.82 ± 31.68	122.00 ± 30.99	146.17 ± 23.81^**^	190.67 ± 21.56^**^
TG (mmol·L^−1^)	0.5–1.0	0.76 ± 0.25	0.56 ± 0.14^*^	0.54 ± 0.17^*^	0.45 ± 0.06^**^
TC (mmol·L^−1^)	1.0–2.0	1.54 ± 0.29	1.59 ± 0.35	1.64 ± 0.43	1.73 ± 0.22
GLU (mmol·L^−1^)	4–8	5.73 ± 1.33	5.59 ± 0.68	5.86 ± 2.01	4.62 ± 0.61
BUN (mmol·L^−1^)	5–10	5.75 ± 2.14	8.30 ± 1.94^**^	7.95 ± 1.58^*^	9.63 ± 1.59^**^
CR (μmol·L^−1^)	25–30	27.02 ± 6.23	25.96 ± 6.94	26.28 ± 6.42	28.80 ± 5.45
UA (μmol·L^−1^)	0.05–0.07	0.061 ± 0.016	0.056 ± 0.011	0.071 ± 0.034	0.063 ± 0.020
LDH (U·L^−1^)	1,200–1,800	1,605.75 ± 278.48	1,802.50 ± 342.99	1,840.88 ± 343.80	1,895.00 ± 195.69
CK (U·L^−1^)	1,100–1,700	1,306.00 ± 309.40	1,547.52 ± 369.63	1,595.46 ± 323.62	1,503.43 ± 192.21

** *p < 0.01 vs. control group*.

* *p < 0.05 vs. control group*.

#### Effect of Oxyclozanide on Relative Organ Weight

During the 28-day experimental period, the mean of relative organ/body ratio (organ weight/body weight × 100%) was statistically decreased in the lung in male rats and increased in the liver in female animals after using high dose of oxyclozanide (*p* < 0.05; Table 3). No statistical (*p* > 0.05) changes in other organs were observed between oxyclozanide treatment and control group (Table 4).

**Table 4:** Effect of oxyclozanide on the body and relative organ weight.

			**Oxyclozanide doses**		
	**Historical controls range**	**0 mg/kg (control)**	**74 mg/kg (low dose)**	**185 mg/kg (medium dose)**	**370 mg/kg (high dose)**
**MALE**					
Initial BW		136.91 ± 8.76	141.08 ± 11.28	147.93 ± 10.05	149.83 ± 7.06
28 days BW		263.01 ± 13.57	259.50 ± 23.02	237.61 ± 19.23	269.23 ± 12.24
Heart	0.3–0.5	0.40 ± 0.055	0.35 ± 0.068	0.35 ± 0.068	0.33 ± 0.061
Liver	3–4	3.60 ± 0.420	3.51 ± 0.612	3.39 ± 0.423	3.46 ± 0.592
Spleen	0.2–0.4	0.26 ± 0.045	0.26 ± 0.063	0.25 ± 0.053	0.24 ± 0.042
Lung	0.5–0.8	0.61 ± 0.097	0.61 ± 0.133	0.65 ± 0.189	0.46 ± 0.128*
Kidney	0.3–0.5	0.38 ± 0.055	0.39 ± 0.095	0.39 ± 0.050	0.39 ± 0.068
Adrenal gland	0.02–0.04	0.02 ± 0.007	0.03 ± 0.012	0.02 ± 0.004	0.02 ± 0.004
Brain	0.6–0.8	0.68 ± 0.109	0.70 ± 0.100	0.71 ± 0.102	0.70 ± 0.123
**FEMALE**					
Initial BW		124.53 ± 9.66	129.67 ± 9.33	127.09 ± 7.46	129.31 ± 10.42
28 days BW		188.79 ± 14.95	191.37 ± 13.95	182.33 ± 10.15	181.31 ± 7.43
Heart	0.3–0.5	0.42 ± 0.068	0.41 ± 0.080	0.36 ± 0.076	0.35 ± 0.071
Liver	3–4	3.30 ± 0.601	3.51 ± 0.616	3.49 ± 0.586	4.00 ± 1.057*
Spleen	0.2–0.4	0.29 ± 0.079	0.28 ± 0.047	0.27 ± 0.044	0.32 ± 0.053
Lung	0.5–0.8	0.70 ± 0.183	0.80 ± 0.186	0.70 ± 0.138	0.74 ± 0.129
Kidney	0.3–0.5	0.42 ± 0.089	0.43 ± 0.077	0.41 ± 0.074	0.45 ± 0.087
Adrenal gland	0.02–0.04	0.03 ± 0.009	0.04 ± 0.009	0.03 ± 0.009	0.03 ± 0.007
Brain	0.6–1.0	0.94 ± 0.147	0.89 ± 0.115	0.94 ± 0.132	0.95 ± 0.163

*BW, body weight*.

* *p < 0.01 vs. control group*.

#### Macroscopic Gross Necropsy

Macroscopic observations showed that no lesions or abnormalities in the architecture of various organs except swelling of kidney occurred more than half in the high-dose group in both sexes. The observations indicated that there was no inflammation, hypertrophy, edema, and atrophy in organ macroscopic examination.

#### Histopathological Analyses

**Figure 2:**
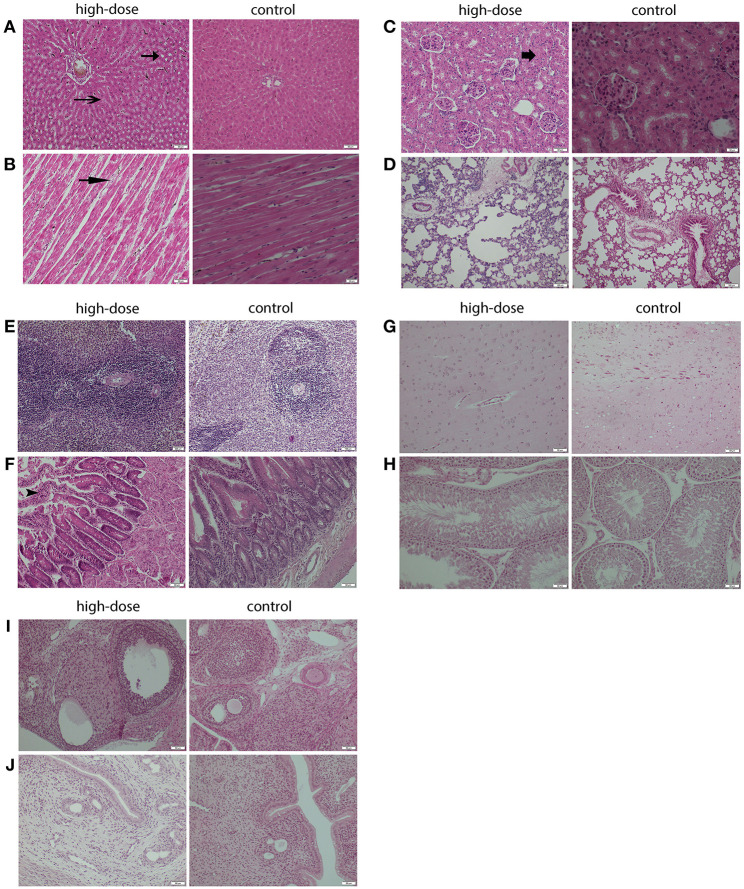
Histopathological analyses of the liver **(A)** and heart **(B)** in high-dose rats with/without oxyclozanide treatment. The arrows indicate that there are granular degeneration and fatty degeneration of liver cells in the liver **(A)** and myocardial fiber degeneration in the heart **(B)**. Histopathological analyses of the kidney **(C)** and lung **(D)** in high-dose rats with/without oxyclozanide treatment. The arrows indicate that there are granular degeneration in renal tubular epithelial cells in the kidney **(C)** and normal in the lung **(D)**. Histopathological analyses of the spleen **(E)** and duodenum **(F)** in high-dose rats with/without oxyclozanide treatment. The arrows indicate that they are normal in the spleen **(E)** and catarrhal enteritis in the duodenum **(F)**. Histopathological analyses of the brain **(G)** and testis **(H)** in high-dose rats with/without oxyclozanide treatment. The arrows indicate that it is normal in the brain **(G)** and testis **(H)**. Histopathological analyses of the ovary **(I)** and uterus **(J)** in high-dose rats with/without oxyclozanide treatment. The arrows indicate that it is normal in the ovary **(I)** and uterus **(J)**.

The histopathological changes could be observed in the heart, liver, kidney, and duodenum of rats treated with the high-dose drug (Figures 2A–**F**) and changes are summarized in Table 5. For instance, myocardial fiber degeneration, myocardial necrosis, and interstitial hydrops could be detected in cardiac tissues having received high or medium dose of oxyclozanide. Glomerulonephritis and granular degeneration of tubular epithelium were shown in renal histology in the high-dose and reversible high-dose groups. Additionally, steatosis and granular degeneration of hepatocytes were observed in the liver in the high-dose group. Compared with the control group, there was any alteration in the brain, lung, and spleen in the treatment groups (Figures 2C–**H**). Moreover, the macroscopic structure of testis, ovary, and uterus was normal in the oxyclozanide treatment groups as compared with the control group (Figures 2G–J).

**Table 5:** Histopathology results of organs in rats treated with oxyclozanide for 28 days.

**Sex/organ**	**Changes**	**Dose**			
		**Control**	**Low**	**Medium**	**High**
**MALE**					
Liver	Granular degeneration	0/12	2/12^c^	7/12^b^	10/12^a^
	Fatty degeneration	0/12	0/12	1/12^a^	4/12^b^
Heart	Myocardial fiber degeneration	0/12	1/12^c^	5/12^b^	10/12^a^
Kidney	Granular degeneration	0/12	3/12^c^	7/12^b^	10/12^a^
Duodenum	Catarrhal enteritis	0/12	0/12	2/12^c^	8/12^b^
**FEMALE**					
Liver	Granular degeneration	0/12	2/12^c^	6/12^b^	10/12^a^
	Fatty degeneration	0/12	0/12	0/12	3/12^b^
Heart	Myocardial fiber degeneration	0/12	1/12^c^	4/12^b^	9/12^a^
Kidney	Granular degeneration	0/12	2/12^c^	5/12^b^	9/12^a^
Duodenum	Catarrhal enteritis	0/10	0/12	4/12^c^	8/12^b^

a *Severe*,

b *Moderate*,

c *Mild*.

*n = 12 photos for each group*.

## Discussion

An acute oral LD_50_ of oxyclozanide in rats was determined to be 3,707 mg/kg. Thus, the design of our current sub-chronic oral toxicity study was based on the LD_50_ dose, and three different doses (370, 175, and 74 mg/kg) representing high, medium, and low dose, respectively, were examined in order to determine a no observed adverse effect level in rats. To our best knowledge, to date, there is no systemic research about subacute toxicity of oxyclozanide to support clinic drug safety. Therefore, in the current study, we performed a series of experiments to study the toxicity and safety of oxyclozanide in Wistar rats.

During the 28-day oral administration of oxyclozanide, no deaths in rats were observed. The results of clinic symptoms showed no obvious changes in general behavior, mucosa color, and posture. However, minor depression and diarrhea were seen in the high and medium dose of oxyclozanide groups only at the first several days of drug administration. This result suggested that the impact of oxyclozanide on depression and diarrhea was short, not permanent. In Walley's research, the toxic signs of oxyclozanide in sheep and cattle are similar to that in rats. Side effects, including diarrhea, loss of appetite, depression, and weight loss, can occur when the dose is as high as 30 mg/kg or higher in sheep and cattle. Weight loss was dramatically reduced and death occurs at a dosage of 60 mg/kg in those animals (7). Additionally, duodenal inflammation was found in animals, which was consistent with our results, indicating that diarrhea symptoms could disturb intestinal function, which is consistent with EMEA's (European Medicines Agency) report (21). In our study, myocardial fibers of rats were damaged, suggesting that oxyclozanide toxicity may cause some damage to the heart. Similarly, blood spots were found in the endocardium of cattle and sheep (7).

The biochemical analyses showed that AST was different between oxyclozanide-treated and untreated control groups. It is known that AST and ALT are released into the serum after relative cells and tissues were damaged (32). In most toxicological studies, the increase of ALT is generally considered to be a practical and specific enzyme indicator of hepatocyte injury. The amount of serum AST may increase during hepatocyte injury. This increase is non-specific because of the high activity of AST in muscle. In liver injury, the AST is often parallel to serum ALT activity. In general, the elevation in AST concentration is related to the number of affected hepatocytes and does not reflect the severity or reversibility of the lesion on a pathological basis. The ALT representing a sensitive indicator in liver damage was not statistically altered during the 28-day study. Thus, the observed AST changes in this study could be associated with some of the other histological changes observed or may be contributed to by multiple tissues. Additionally, oxyclozanide was reported to cause ALP increase and toxic effects on the liver in experimental rats (7). The increase of ALP activity is an indication of cholestasis, which may be secondary to intrahepatic bile duct obstruction or extrahepatic obstruction caused by hepatocyte swelling. Drug-induced hepatocytes necrosis can also increase the activity of ALP (33). In this test, the level of ALP in the high- and medium-dose groups was statistically elevated (*p* < 0.01). These results indicate that oxyclozanide may bring liver toxicity. It reported that the level of TBIL, a breakdown product of normal haem catabolism, is an indicator for estimating liver function and cholestasis (34). Pathological reductions in TBIL are common in anemia caused by chronic glomerulonephritis (35) or aplastic anemia (36), but are not common in toxicological studies and do not have sufficient hematological changes to support this viewpoint. BUN is a kind of marker for assessing renal function in clinic diagnosis (32). During the study, the level of BUN increased in the high-dose oxyclozanide group in both males and females, which indicates that there was damage for functional nephrons because of inflammation or toxin productions in the kidney. Moreover, the level of UA was increased in males in the high-dose group, which may imply the oxyclozanide has an influence on renal function (32). Compared with the control values, the levels of LDH and CK were elevated in the treated group, which suggest that there were muscle damages, especially in liver and heart (32). The liver and kidney are important and sensitive organs to drug actions (37). Specifically, the liver is an organ for the metabolism of cholesterol and glucose and the kidney was the main organ for excretion (38). The statistical elevation (*p* < 0.05 or *p* < 0.01) of BUN levels and the histopathology results with alternation of functional nephrons suggested that oxyclozanide could damage renal function.

During the histopathological examination of cardiac tissues, we detected some histopathological findings as evidence of toxicity like fiber degeneration, necrosis, and interstitial hydrops, which indicated that there were toxic damages after drug administration. The histopathological study also showed that glomerulonephritis and granular degeneration of tubular epithelium have alternated in renal function, which implied that oxyclozanide produced toxicity in the renal system. Steatosis and granular degeneration were found in the liver tissue of rats after administration, suggesting that there were toxic lesions in the liver. In the previous report, during the 90-day subchronic oral toxicity study, the brain had vacuolization phenotype in a high-dose oxyclozanide group in rats and dogs (21). However, our histopathological studies showed no pathological changes in brains between the treatment and the controls. It suggests that oxyclozanide doesn't show damage to the nervous system under subacute exposure. Besides, the histology of duodenum showed that there was catarrhal enteritis in the normal architecture resulting from diarrhea after administration.

The uncoupling effect of oxidative phosphorylation from drugs had a pharmacological effect on parasite. As a famous drug of salicylanilides, niclosamide has been considered to have an excellent safety range for a very long time. LD_50_ of niclosamide was 2,500 mg/kg in rats (39) and 1,000 mg/kg in mice (40). The maximum tolerable dose of niclosamide was 2,000 mg/kg during the 28-day toxicity study of oral administration in rats. Moreover, many researchers have confirmed that niclosamide brought developmental and genetic toxicity to animals (41–44). However, there was still no related report on oxyclozanide.

## Conclusion

The LD_50_ of oxyclozanide was 3,707 mg/kg BW for experimental rats in both sex in the acute study. During the 28-day administration, differences in the histopathological, hematological, and biochemical assays were found at the 185- and 370-mg/kg dose groups vs. the untreated control group. These results suggest that oxyclozanide could cause damage in the liver, kidney, and heart. According to those results, the LOAEL level was 74 mg/kg for rats during the 28-day toxicity study, which provides basis for clinic use of oxyclozanide and for determining a reasonable safe dose.

## Data Availability

The datasets generated for this study are available on request to the corresponding author.

## Ethics Statement

The animal study was reviewed and approved by Institutional Animal Care and Use Committee of Lanzhou Institute of Husbandry and Pharmaceutical Science of Chinese Academy of Agricultural Sciences.

## Author Contributions

JiyZ, WW, and ZD conceived and designed the experiments. XZ, XW, ZD, and WW performed the experiments. ZD, JilZ, and BL analyzed the data. ZD, FC, and WW interpreted the references and provided background information. WW and ZD wrote the paper.

### Conflict of Interest Statement

The authors declare that the research was conducted in the absence of any commercial or financial relationships that could be construed as a potential conflict of interest.
