# Corrigendum: Acute and subacute toxicity assessment of oxyclozanide in Wistar rats

**DOI:** 10.3389/fvets.2023.1130466

**Published:** 2023-02-01

**Authors:** Weiwei Wang, Zhen Dong, Jili Zhang, Xuzheng Zhou, Xiaojuan Wei, Fusheng Cheng, Bing Li, Jiyu Zhang

**Affiliations:** ^1^Key Laboratory of New Animal Drug Project of Gansu Province, Lanzhou, China; ^2^Key Laboratory of Veterinary Pharmaceutical Development, Ministry of Agriculture, Lanzhou, China; ^3^Lanzhou Institute of Husbandry and Pharmaceutical Sciences, Chinese Academy of Agricultural Sciences, Lanzhou, China

**Keywords:** oxyclozanide, acute, subacute, toxicity, rats

In the published article Reference Manual of Laboratory Animal Blood Physiology and Biochemistry (*book*) was not cited in the article. The citation has now been inserted in Tables 2, 3, [Historical controls range] and should read:

**Table 2:** Haematological values of rats in oxyclozanide-treated group and control group.

	**Historical controls range (45)**	**Oxyclozanide doses (mg/kg)**			
		**0 mg/kg** **(control)**	**74 mg/kg** **(low-dose)**	**185 mg/kg** **(medium-dose)**	**370 mg/kg** **(high-dose)**
**Male**					
WBC (× 10^9^·L^−1^)	4–6	5.28 ± 2.12	4.00 ± 1.73	5.47 ± 2.44	5.13 ± 1.22
LYM (× 10^9^·L^−1^)	2–5	2.87 ± 1.70	3.50 ± 1.22	4.10 ± 2.01	3.88 ± 0.96
Mon (× 10^9^·L^−1^)	0.05–0.10	0.05 ± 0.071	0.06 ± 0.074	0.08 ± 0.071	0.08 ± 0.044
Gran (× 10^9^·L^−1^)	0.5–1.0	0.54 ± 0.46	0.63 ± 0.43	0.80 ± 0.48	0.98 ± 0.48
RBC (× 10^12^·L^−1^)	6–8	7.04 ± 0.38	7.29 ± 0.51	7.03 ± 0.48	6.93 ± 0.45
HGB (g·L −1)	120–140	137.55 ± 5.47	128.60 ± 22.83	135.38 ± 10.21	136.17 ± 10.53
HCT	35–45	41.57 ± 1.50	43.14 ± 3.10	41.26 ± 3.02	41.18 ± 3.11
MCV (fL)	55–65	59.16 ± 1.91	59.24 ± 0.97	58.75 ± 1.88	59.47 ± 2.32
MCH (pg)	17–21	19.51 ± 0.48	17.37 ± 3.01^**^	19.18 ± 0.46	19.58 ± 0.67
MCHC (g·L^−1^)	270–340	330.36 ± 4.39	293.67 ± 50.54	327.50 ± 7.44	330.17 ± 7.98
RDW	10–15	11.60 ± 2.21	10.84 ± 1.69	12.75 ± 1.84	12.60 ± 1.81
PLT (× 10^9^·L^−1^)	800–1,000	860.18 ± 153.11	939.44 ± 154.04	956.38 ± 68.66	759.40 ± 144.87
MPV (fL)	5–7	6.38 ± 0.25	6.28 ± 0.31	6.09 ± 0.50	5.95 ± 0.66
PDW	15–17	16.19 ± 0.18	16.29 ± 0.32	16.13 ± 0.25	16.22 ± 0.43
PCT (ng·L^−1^)	0.4–0.8	0.53 ± 0.079	0.56 ± 0.073	0.58 ± 0.052	0.58 ± 0.090
**Female**					
WBC (× 10^9^·L^−1^)	4–6	4.14 ± 2.16	5.76 ± 1.63	4.66 ± 1.98	5.01 ± 1.36
LYM (× 10^9^·L^−1^)	3–5	3.46 ± 1.55	4.64 ± 0.612	4.25 ± 1.09	4.11 ± 1.14
Mon (× 10^9^·L^−1^)	0.05–0.10	0.09 ± 0.074	0.10 ± 0.076	0.08 ± 0.067	0.06 ± 0.052
Gran (× 10^9^·L^−1^)	0.6–1.1	0.86 ± 0.45	0.90 ± 0.41	1.00 ± 0.42	0.92 ± 0.20
RBC (× 10^12^·L^−1^)	6–8	7.45 ± 0.21	7.21 ± 1.31	7.26 ± 1.47	7.23 ± 0.35
HGB (g·L^−1^)	120–140	148.45 ± 7.85	126.38 ± 28.23	136.80 ± 38.84	143.88 ± 6.94
HCT	35–45	44.50 ± 1.22	42.80 ± 7.84	42.99 ± 8.63	43.70 ± 2.07
MCV (fL)	60 ± 6	59.86 ± 1.73	53.05 ± 19.12	59.30 ± 1.00	60.55 ± 2.37
MCH (pg)	17–21	19.89 ± 0.81	17.53 ± 2.35^*^	18.44 ± 3.49	19.88 ± 0.69
MCHC (g·L^−1^)	270–340	333.00 ± 15.50	296.13 ± 41.19^*^	311.78 ± 58.95	328.88 ± 4.12
RDW	10–15	14.92 ± 0.45	13.80 ± 1.16^**^	14.78 ± 0.55	14.41 ± 0.77
PLT (× 10^9^·L^−1^)	900–1,100	1,015.82 ± 91.63	970.38 ± 215.43	921.38 ± 192.32	953.33 ± 155.58
MPV (fL)	5–7	6.25 ± 0.22	6.33 ± 0.26	6.46 ± 0.69	6.18 ± 0.37
PDW	15–17	16.07 ± 0.13	16.13 ± 0.18	16.50 ± 1.15	16.10 ± 0.41
PCT (ng·L^−1^)	0.4–0.8	0.63 ± 0.056	0.55 ± 0.132^*^	0.49 ± 0.174	0.50 ± 0.197

* *p* < 0.05 vs. control group.

** *p* < 0.01 vs. control group.

**Table 3:** Liver function related to biochemical profiles.

	**Historical controls range (45)**	**Oxyclozanide doses**			
		**0 mg/kg** **(Control)**	**74 mg/kg** **(Low-dose)**	**185 mg/kg** **(Medium-dose)**	**370 mg/kg** **(High-dose)**
**Male**					
TBIL (μmol·L^−1^)	0.3–0.7	0.52 ± 0.23	0.54 ± 0.29	0.39 ± 0.19	0.35 ± 0.17
DBIL (μmol·L^−1^)	0.8–1.4	1.20 ± 0.55	0.97 ± 0.32	1.01 ± 0.22	1.32 ± 0.51
TP (g·L^−1^)	30–50	41.94 ± 15.15	36.17 ± 11.22	41.83 ± 9.38	49.97 ± 8.13
ALB (g·L^−1^)	18–26	19.45 ± 6.61	18.30 ± 5.53	19.63 ± 4.04	24.36 ± 3.48^*^
GLO (g·L^−1^)	16–26	22.45 ± 8.63	17.87 ± 5.73	22.20 ± 5.57	25.62 ± 4.90
ALT (U·L^−1^)	25–35	34.88 ± 12.47	27.35 ± 9.97	34.62 ± 9.68	32.45 ± 5.23
AST (U·L^−1^)	95–150	98.43 ± 33.86	132.14 ± 35.72	158.11 ± 48.63^**^	199.45 ± 32.55^**^
ALP (U·L^−1^)	120–200	131.71 ± 25.22	135.00 ± 26.93	197.00 ± 46.29^*^	224.00 ± 81.28^**^
TG (mmol·L^−1^	0.5–.07	0.62 ± 0.18	0.63 ± 0.17	0.68 ± 0.33	0.56 ± 0.16
TC (mmol·L^−1^	1.0–2.0	1.52 ± 0.47	1.34 ± 0.45	1.70 ± 0.36	1.70 ± 0.36
GLU (mmol·L^−1^)	4–8	6.64 ± 1.50	6.00 ± 1.15	5.49 ± 1.45	4.95 ± 1.09^**^
BUN (mmol·L^−1^)	5–9	5.49 ± 1.34	7.01 ± 1.98	7.17 ± 1.33	8.76 ± 2.99^**^
CR (μmol·L^−1^)	20–30	24.89 ± 17.67	21.05 ± 7.06	25.22 ± 5.19	29.46 ± 8.73
UA (μmol·L^−1^)	0.04–0.06	0.046 ± 0.012	0.045 ± 0.017	0.065 ± 0.021	0.080 ± 0.048^*^
LDH (U·L^−1^)	1,200–2,000	1,320.30 ± 469.50	1,582.11 ± 303.31	1,864.00 ± 433.37^**^	1,954.77 ± 236.24^**^
CK (U·L^−1^)	1,100–1,700	1,188.72 ± 488.39	1,210.48 ± 279.84	1,499.38 ± 405.02	1,628.94 ± 312.20^**^
**Female**					
TBIL (μmol·L^−1^)	0.4–0.8	0.69 ± 0.27	0.45 ± 0.26^*^	0.49 ± 0.14	0.27 ± 0.12^**^
DBIL (μmol·L^−1^)	1–2	1.32 ± 0.42	1.27 ± 0.47	1.46 ± 0.49	1.48 ± 0.43
TP (g·L^−1^)	40–48	45.85 ± 9.98	42.24 ± 10.40	45.40 ± 13.24	40.32 ± 4.21
ALB (g·L^−1^)	20–26	23.33 ± 4.65	21.60 ± 5.10	22.49 ± 6.09	25.87 ± 3.98
GLO (g·L^−1^)	20–30	22.52 ± 5.34	20.65 ± 5.36	22.91 ± 7.30	28.97 ± 6.49^**^
ALT (U·L^−1^)	25–35	26.11 ± 7.17	25.64 ± 10.56	30.68 ± 11.99	33.92 ± 6.11
AST (U·L^−1^)	130–180	137.10 ± 31.18	155.70 ± 39.79	177.71 ± 49.51^*^	206.60 ± 25.16^**^
ALP (U·L^−1^)	90–140	97.82 ± 31.68	122.00 ± 30.99	146.17 ± 23.81^**^	190.67 ± 21.56^**^
TG (mmol·L^−1^)	0.5–1.0	0.76 ± 0.25	0.56 ± 0.14^*^	0.54 ± 0.17^*^	0.45 ± 0.06^**^
TC (mmol·L^−1^)	1.0–2.0	1.54 ± 0.29	1.59 ± 0.35	1.64 ± 0.43	1.73 ± 0.22
GLU (mmol·L^−1^)	4–8	5.73 ± 1.33	5.59 ± 0.68	5.86 ± 2.01	4.62 ± 0.61
BUN (mmol·L^−1^)	5–10	5.75 ± 2.14	8.30 ± 1.94^**^	7.95 ± 1.58^*^	9.63 ± 1.59^**^
CR (μmol·L^−1^)	25–30	27.02 ± 6.23	25.96 ± 6.94	26.28 ± 6.42	28.80 ± 5.45
UA (μmol·L^−1^)	0.05–0.07	0.061 ± 0.016	0.056 ± 0.011	0.071 ± 0.034	0.063 ± 0.020
LDH (U·L^−1^)	1,200–1,800	1,605.75 ± 278.48	1,802.50 ± 342.99	1,840.88 ± 343.80	1,895.00 ± 195.69
CK (U·L^−1^)	1,100–1,700	1,306.00 ± 309.40	1,547.52 ± 369.63	1,595.46 ± 323.62	1,503.43 ± 192.21

** *p* < 0.01 vs. control group.

* *p* < 0.05 vs. control group.

“[Historical controls range (45)]”

45. Wang D, Zeng L, Shang S. *Reference Manual of Laboratory Animal Blood Physiology and Biochemistry*. Science Publishing (2011).

In the published article, there was an error in Table 2 as published. The units for the hematological parameters used in Table 2 were missing. The corrected Table 2 and its caption ^**^Hematological values of rats in the oxyclozanide-treated group and the control group appear below.

In the published article, there was an error in Table 3 as published. The units for the biochemical parameters used in Table 3 were missing. The corrected Table 3 and its caption ^**^Liver function related to biochemical profiles appear below.

In the published article, **Supplementary Appendix 1** was mistakenly not included in the publication. The missing material appears below:

The blood samples collected in the EDTAK2-coated tubes were analyzed using Mindray BC-2800 Vet Automatic blood analyser (MINDRAY Medical International Co., Ltd). Erba XL-640 Automatic biochemical analyser (Erba Mannheim Co., Ltd.) was used for biochemical examination.

The authors apologize for this error and state that this does not change the scientific conclusions of the article in any way. The original article has been updated.

