# Supplementary material for: Acute and Subacute Toxicity Assessment of Oxyclozanide in Wistar Rats
Source: Front Vet Sci. 2019 Sep 6;6:294. doi: 10.3389/fvets.2019.00294 (PMC6743374; doi:10.3389/fvets.2019.00294)
Supplement: Supplementary file 1 [file Table_1.docx]

The blood samples collected in the EDTAK2-coated tubes were analyzed using Mindray BC-2800 Vet Automatic blood analyser (MINDRAY Medical International Co., Ltd). Erba XL-640 Automatic biochemical analyser (Erba Mannheim Co., Ltd.) was used for biochemical examination.
